# Preventing Respiratory Viral Illness Invisibly (PRiVII): protocol for a pragmatic cluster randomized trial evaluating far-UVC light devices in long-term care facilities to reduce infections

**DOI:** 10.1186/s13063-024-07909-0

**Published:** 2024-01-26

**Authors:** Hayden P. Nix, Samantha Meeker, Caroline E. King, Melissa Andrew, Ian R. C. Davis, Prosper S. Koto, Meaghan Sim, Jennifer Murdoch, Glenn Patriquin, Chris Theriault, Stephanie Reidy, Michael Rockwood, Tara Sampalli, Samuel D. Searle, Kenneth Rockwood

**Affiliations:** 1 Geriatric Medicine Research, Halifax, NS Canada; 2 Department of Medicine, Dalhousie University, Halifax, NS Canada; 3 Research, Innovation and Discovery, Nova Scotia Health, Halifax, NS Canada; 4 Division of Geriatric Medicine, Dalhousie University, Halifax, NS Canada; 5 Division of Infectious Diseases, Department of Medicine, Nova Scotia Health, Halifax, NS Canada; 6 Department of Pathology, Faculty of Medicine, Dalhousie University, Halifax, NS Canada; 7 Research Methods Unit, Nova Scotia Health, Halifax, NS Canada; 8 Division of Microbiology, Department of Pathology and Laboratory Medicine, Nova Scotia Health, Halifax, NS Canada; 9 Division of Rheumatology, Nova Scotia Health, Halifax, NS Canada; 10 Medical Research Council Unit for Lifelong Health and Ageing at University College London, University College London, London, UK; 11 Frailty & Elder Care Network, Nova Scotia Health, Halifax, NS Canada

**Keywords:** Respiratory viral illness, Long-term care, Cluster randomized controlled trial, Geriatrics

## Abstract

**Background:**

Respiratory viral illness (RVI)—e.g., influenza, COVID-19—is a serious threat in long-term care (LTC) facilities. Standard infection control measures are suboptimal in LTC facilities because of residents’ cognitive impairments, care needs, and susceptibility to loneliness and mental illness. Further, LTC residents living with high degrees of frailty who contract RVIs often develop the so-called atypical symptoms (e.g., delirium, worse mobility) instead of typical cough and fever, delaying infection diagnosis and treatment. Although far-UVC (222 nm) light devices have shown potent antiviral activity in vitro, clinical efficacy remains unproven.

**Methods:**

Following a study to assay acceptability at each site, this multicenter, double-blinded, cluster-randomized, placebo-controlled trial aims to assess whether far-UVC light devices impact the incidence of RVIs in LTC facilities. Neighborhoods within LTC facilities are randomized to receive far-UVC light devices (222 nm) or identical placebo light devices that emit only visible spectrum light (400–700 nm) in common areas. All residents are monitored for RVIs using both a standard screening protocol and a novel screening protocol that target atypical symptoms. The 3-year incidence of RVIs will be compared using intention-to-treat analysis. A cost-consequence analysis will follow.

**Discussion:**

This trial aims to inform decisions about whether to implement far-UVC light in LTC facilities for RVI prevention. The trial design features align with this pragmatic intent. Appropriate additional ethical protections have been implemented to mitigate participant vulnerabilities that arise from conducting this study. Knowledge dissemination will be supported through media engagement, peer-reviewed presentations, and publications.

**Trial registration:**

ClinicalTrials.gov NCT05084898. October 20, 2021.

**Supplementary Information:**

The online version contains supplementary material available at 10.1186/s13063-024-07909-0.

## Introduction

### Background and rationale {6a}

For decades, respiratory viral illness (RVI) has been recognized as a serious hazard for long-term care (LTC) facility residents [1]. The COVID-19 pandemic has underscored this vulnerability; LTC residents account for over 40% of all COVID-19 deaths in Canada [2].

Standard infection control interventions, including handwashing, physical distancing, and personal protective equipment [3, 4], have proved to be suboptimal in LTC for several reasons. First, dementia and frailty can impair adherence to handwashing and physical distancing recommendations. Second, many residents need intimate personal care, thereby obliging close proximity [5–7]. Third, physical distancing can exacerbate residents’ susceptibility to loneliness and mental illness [8, 9].

Far-UVC light (222 nm) could help to address the shortcomings of standard infection control interventions. Far-UVC light can kill a variety of microbes in vitro, including influenza viruses and SARS-CoV-2 [10–12], without damaging human tissues [13–15]. Further, far-UVC light can effectively kill airborne microbes in a full-sized room, even with continuous introduction of microbes into the space [16]. As this intervention does not require active adherence, it could provide a valuable layer of protection of LTC residents. Despite mounting evidence of in vitro efficacy, to our knowledge, no clinical trials have been conducted to evaluate the effectiveness of far-UVC at reducing the incidence of RVIs in clinical settings, including LTC facilities.

Capturing all RVIs will be important for determining the efficacy of far-UVC light in this trial. For this reason, it is important to recognize that RVIs, including COVID-19, often present “atypically” in frail older adults [17, 18]. Illness in these patients may present with delirium, but without associated cough or fever [18–20]. In short, an “atypical presentation” in many people is how illness typically presents in older adults who live with frailty [21]. Atypical presentations can cause standard screening protocols to yield false negatives, delaying diagnosis, treatment, and more stringent infection control interventions (e.g., isolation). To capture RVIs as early as possible in the trial, three additional screening tools: the single question in delirium (SQiD), the single question in reduced mobility (SQiRM), and the single question in function (SQiF) will be used. The SQiD asks: “Is the patient more confused than before?” [22] and has been validated in clinical settings [22–24]. Building on the principles of the SQiD, the SQiRM and the SQiF evaluate changes in mobility and overall function, respectively. SQiRM and SQiF are novel screening tools.

### Objectives {7}

The Preventing Respiratory Viral Illness Invisibly (PRiVII) trial is a pragmatic cluster randomized trial that aims to answer the following question: Should far-UVC light be used in LTC facilities to help reduce the incidence of RVIs? The trial has completed Phase 1. This article describes the protocol for Phase 2 of the trial, which includes updated far-UVC light devices and a third LTC facility study site. Prior to the trial, stakeholder engagement was conducted to ensure that study procedures were compatible with the LTC facility setting and to promote the autonomy, welfare, and justice interests of participants and associated parties [25].

### Trial design {8}

This is a multicenter, double-blinded, cluster randomized, placebo-controlled, superiority trial with two parallel arms. The protocol is registered on ClinicalTrials.gov: registration number NCT05084898. It has pragmatic intent; it aims to inform clinical decisions of whether far-UVC light should be implemented in LTC facilities to reduce the incidence of RVIs. Cluster randomization is necessary because placing far-UVC lights in common areas constitutes a cluster-level intervention.

## Methods

### Study setting {9}

**Fig. 1:**
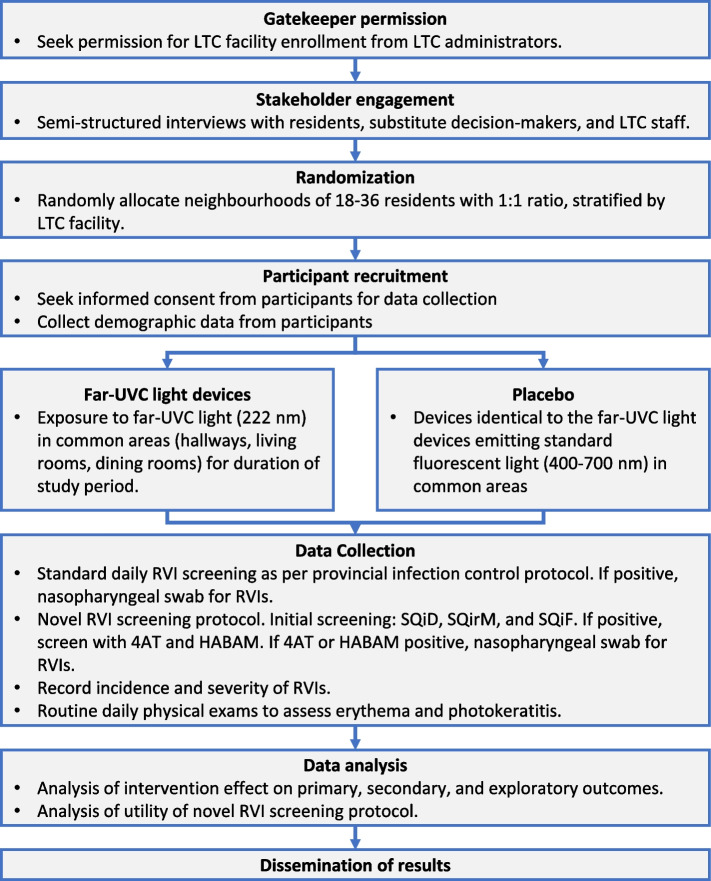
Trial flow chart

Two LTC facilities in Nova Scotia were included in Phase 1 of the trial. In Phase 2, a third LTC facility was added. The trial flow chart is reported in Fig. 1.

In Phase 1 of the trial, two LTC facilities in Nova Scotia were selected: one in Halifax and one in Falmouth. These locations vary in many aspects, including building type, layout, and size. The layout of the Halifax LTC facility is similar to an acute care ward and has semi-private rooms (private bedroom with shared bathrooms) and shared bedrooms (shared by two residents), whereas the layout of the Falmouth LTC facility is similar to a large house and has private bedrooms and private bathrooms. These sites were selected for the study as a result of feasibility and logistical constraints. There are four participating neighbourhoods at the Halifax LTC facility and two participating neighbourhoods at the Falmouth LTC facility. There are 18–36 residents living in each neighborhood.

In Phase 2, a LTC facility in Cape Breton, Nova Scotia, was added to the trial. It has private bedrooms with private bathrooms and semi-private rooms. There are five participating neighborhoods in this LTC facility.

### Eligibility criteria {10}

All residents in participating neighborhoods in each LTC facility are eligible for study participation. In Nova Scotia, people may only move into LTC facilities if they are medically stable and have nursing needs that cannot be met through home care. Therefore, frailty and dementia are highly prevalent in LTC facilities [26]. LTC facility staff are ineligible to participate because they routinely travel between neighborhoods within LTC facilities and therefore cannot be randomized. LTC facility visitors are ineligible because of the likelihood of exposure to RVIs outside of the LTC facilities.

### Who will take informed consent {26a}

Research nurses at each LTC facility seek informed consent from all LTC facility residents or their substitute decision-maker (SDM) for study data collection.

### Additional consent provisions for collection and use of participant data and biological specimens {26b}

Participant data will not be used in ancillary studies.

## Interventions

### Explanation for choice of comparator {6b}

LTC facility neighborhoods randomized to the control arm receive standard disinfection procedures and are subject to Nova Scotia’s mandated COVID-19 prevention measures and infection prevention and control guidelines. These consist in personal protection equipment, cleaning and sanitizing, physical distancing, and restrictions on visitors and nonessential personnel [27]. These guidelines fluctuate depending on COVID-19 outbreaks in the province and are expected to vary during the study. Placebo light devices that are identical to the far-UVC light devices but emit only visible spectrum light (400–700 nm) are installed in common areas (hallways, living rooms, and dining rooms). The clinical effectiveness of the intervention has not been proven in previous clinical trials; therefore, a placebo control—in combination with usual care infection control measures—is justified.

### Intervention description {11a}

LTC facility neighborhoods randomized to the intervention arm receive far-UVC light devices in addition to mandated infection control interventions. The far-UVC light devices are approximately the size of a smoke detector. They are installed on the ceiling in high traffic common areas in LTC facilities, where residents spend approximately 3 to 4 h per day. The lights are on 24 h per day throughout the data collection period of the study. The devices produce no heat, and the only indicators of operation are an indicator LED and a dim blue light from a square in the center.

At the onset of Phase 1, the threshold limit value (TLV) set by the American Conference of Governmental Industrial Hygienists (ACGIH) for 222-nm far-UVC was 23 mJ/cm^2^ per 8-h working day. The far-UVC devices used in Phase 1 emit 222-nm light, at an 80° beam angle, with an output tuned to meet this TLV at a height of 2.1 m from the floor for a standard 2.7-m ceiling. Since the start of Phase 1, ACGIH has updated 222-nm far-UVC TLVs to 160 mJ/cm^2^ per 8 h for eye exposure and 479 mJ/cm^2^ per 8 h for skin exposure [28].

The American National Standards Institute (ANSI) and Illuminating Engineering Society (IES) have adopted ACGIH’s new TLVs in their ANSI/IES RP-27.1–22 standard. To reflect these updated photobiological limits, the far-UVC devices will be replaced in Phase 2. The new devices (1) emit higher irradiance to achieve higher doses than Phase 1, (2) are equipped with an optical filter to attenuate UVC wavelengths above 23 nm, and (3) are equipped with a diffuser to increase the beam angle to 108°.

### Criteria for discontinuing or modifying allocated interventions {11b}

Far-UVC light in common areas is a cluster-level intervention. Therefore, it is infeasible to modify it for an individual participant. Based on previous studies, it is anticipated to pose minimal risk to participants.

### Strategies to improve adherence to interventions {11c}

Far-UVC light does not require participants to actively adhere. Participants will be free to use common areas as much as they wish, in keeping with the trial’s pragmatic intent.

### Relevant concomitant care and interventions that are permitted or prohibited during the trial {11d}

Throughout the trial, participants in the intervention and control arm all receive usual care, including standard disinfection control measures.

### Provision of posttrial care {30}

After the trial, participants will continue to receive usual care.

### Outcomes {12}

The primary outcome is the incidence of RVIs, diagnosed from nasopharyngeal swabs. RVIs include SARS-CoV-2, influenza A, influenza B, and other upper respiratory viruses, depending on epidemiology and mandated testing protocols.

Secondary outcomes include the incidence of erythema and photokeratitis among LTC residents; the sensitivity, specificity, positive predictive value, negative predictive value, and validity of SQiD, SQiRM, and SQiF for identifying RVIs, and the value for money for the implementation of the far-UVC infection control strategies. The SQiD has been validated as a screening tool for delirium in geriatric inpatients and oncology inpatients [23, 24]. It has not been validated in LTC homes. The SQiRM and the SQiF have not been validated—they will be assessed for the first time in this trial.

Exploratory outcomes include the severity of RVI (oxygen requirements, recovery rates, and time to recovery or death). The outcomes in Phase 2 are unchanged from Phase 1.

### Participant timeline {13}

LTC facility residents are approached by research staff to inform them about the trial and seek consent for data collection. For residents living in the LTC facilities prior to study onset, this occurred before the onset of the trial. Whenever a new individual moves into a participating LTC home, they will be approached shortly after moving in. Residents undergo daily assessments as part of routine care, and their data is collected by nurses working in the facility. The timeline is summarized in Table 1.

**Table 1:**
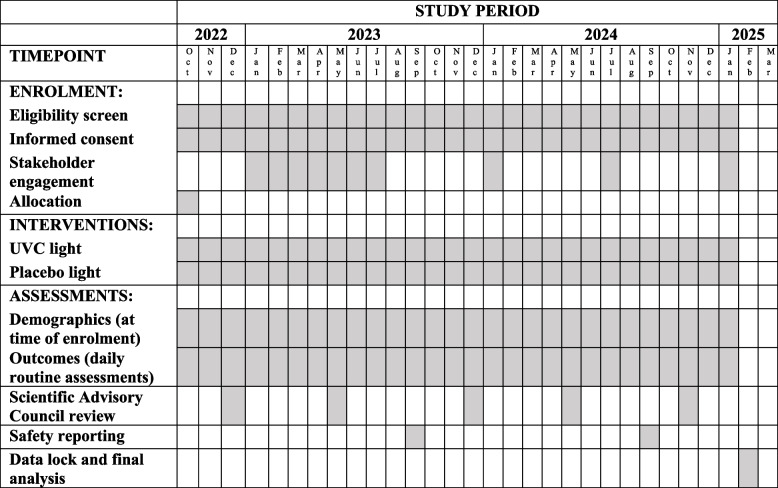
SPIRIT diagram

### Sample size {14}

Prior to Phase 1, sample size estimations were limited by uncertainty surrounding the expected incidence of SARS-CoV-2 in the study setting. The approach taken here was to include as many clusters as feasibly possible given budget and logistical constraints, and then estimate the necessary sample size 1 year into the study.

The sample size was determined using a log-rank test comparing two survival rates in a cluster randomized design. The intra-class correlation coefficient (ICC) for the neighborhoods and the survival probability for the entire sample was calculated using the data collected by year 1. The survival probability should be calculated for just the control group; however, using the entire sample enabled the primary biostatistician to remain blinded, which is the recommended practice [29–31]. Theoretically, this approach would lead us to overestimate the necessary sample (if we assume the treated group had less or equal cases to the control group).

At the time of the interim analysis, there were 178 participants in the trial (20–25 per cluster), and the median time in the study was 0.98 years (*IQR*: 0.49–1.02). There were 30 cases resulting in an incidence rate of 0.22 (95% *CI*: 0.15–0.31). The ICC was estimated using a random effects logistic regression model [32], but the low incidence rate meant the estimate was not reliable (*ICC* = 0.12, 95% *CI*: 0.002–0.883). We believe that our incidence rate estimate of 0.22 cases per year is low and unlikely to represent the future rates of infection because of the changes in prevention strategies since the first year of data collection. We have therefore modelled several scenarios where we vary the survival probability between 0.4 and 0.7 (Additional File 1).

The fixed parameters used were power at 0.8, alpha at 0.05, the number of clusters in the experimental and placebo groups (3 in control and 3 in the treatment), and a hazard ratio of 0.7. Analysis was completed using the Stata’s *power logrank, cluster* command. We attempted to include varying levels of ICC; however, our sample size estimates were very sensitive to the ICC and could not actually be estimated with our parameters specified above. Without accounting for the ICC, we would require between 470 and 968 participants if the survival probability in the control group was 0.4 or 0.7 respectively. At the time of this calculation, we anticipated recruiting a maximum of 250 participants within our timeline, which would likely leave us underpowered. Based on these results, we added an additional site (five more clusters) and extended the study by a year (i.e., Phase 2). With these modifications, we anticipate being able to recruit approximately 500 persons into the study. We will conduct another interim analysis at year 2 of the study to determine if these modifications will suffice. At that time, we anticipate being able to define our survival probability more accurately and properly account for the ICC in the sample size calculation.

### Recruitment {15}

To recruit participants, all LTC facility residents or their SDMs are approached by research nurses at each LTC facility.

## Assignment of interventions: allocation

### Sequence generation {16a}

Randomization is at the level of “neighborhoods” within LTC facilities (distinct areas within LTC facilities that house social groups of 18–36 residents). In Phase 1, neighborhoods in the Halifax and Falmouth LTC facilities, 16 in total, were randomized with a 1:1 allocation ratio. In Phase 2, five neighborhoods were randomized in the Cape Breton LTC facility: two to the placebo arm and three to the active arm. Randomization is stratified by LTC facility. Randomization was performed by a blinded biostatistician who is otherwise uninvolved with the study using SAS 9.4 software. 

### Concealment mechanism {16b}

The biostatistician communicated the neighborhood assignment to the sole unblinded study coordinator, who then facilitated the installation of placebo and intervention light devices.

### Implementation {16c}

The biostatistician generated the allocation sequence and communicated it to the unblinded study coordinator. The unblinded study coordinator then worked with blinded LTC facility administrators to coordinate the installation of the UVC and placebo light devices.

## Assignment of interventions: blinding

### Who will be blinded {17a}

The far-UVC lights are indistinguishable from the placebo lights. LTC facility residents, LTC facility staff, on-site researchers, and the biostatistician performing the analysis are blinded to group allocation. Only two members of the research team are unblinded to coordinate light installation by an independent third-party contractor. Blinded team members do not have access to participants’ identifiable information.

### Procedure for unblinding if needed {17b}

Emergency envelopes containing the randomization allocation are stored in two locations: a locked drawer in the study coordinator’s office and the unblinded study coordinator carries the second envelope on her during work hours. In case of serious adverse event, the study coordinators are able to facilitate unblinding.

## Data collection and management

### Plans for assessment and collection of outcomes {18a}

Participant information, including demographics (e.g., age, sex, ethnicity), health status (e.g., presence of chronic diseases, a Frailty Index calculated from the resident Comprehensive Geriatric Assessments routinely completed twice a year [33], smoking status, verbal/non-verbal), vaccination status for influenza and COVID-19, and emotional/social support (number of visitors, time spent telecommunicating with family or friends) are collected from medical records and a baseline history and physical exam conducted by research nurses at the time of enrollment. Residents in participating neighborhoods who do not consent to data collection are included from RVI case counts, and only their neighborhood, age, and sex are collected from their medical records.

The novel screening protocol is administered daily to residents in both study arms. This includes assessment using the SQiD, SQiRM, and SQiF in addition to the standard screening protocol for infectious symptoms. The SQiD, SQiRM, and SQiF are single question assessments with binary outcomes. The SQiD asks: “Is the patient more confused than before?” The SQiRM asks: “Is the person’s mobility worse (reduced) compared with before (in the last 24 hours)?” and the SQiF asks: “Is the person’s functioning worse than before (in the last 24 hours)?” Compared to a psychiatric interview, the SQiD has a sensitivity of 80% (95% CI 28.3– 99.49%), a specificity of 71% (41.90–91.61%), a positive predictive value of 50%, and a negative predictive value of 91% (58.72–99.77%) [22]. It has been validated in geriatric inpatients and oncology inpatients but has not been validated in LTC homes. The SQiRM and SQiF will be evaluated for the first time in this trial.

If any of the SQiD, SQiRM, or SQiF yield a positive finding, then the resident is assessed with the 4 A’s test (4AT) and Hierarchical Assessment of Balance and Mobility (HABAM) screening every day for 4 days or until a new stable baseline is established (defined as consistent 4AT and HABAM for 7 consecutive days). If positive on 4AT, HABAM, or standard screening protocol, the resident receives a nasopharyngeal swab for respiratory viruses.

In this trial, the 4AT and HABAM are used as process measures to help evaluate the clinical utility of the SQiD, SQiRM, and SQiF. The 4AT is a screening tool for delirium, typically used in conjunction with the SQiD. It requires the assessor to evaluate the patient’s alertness and the acuity of the change in mental status, and ask questions that evaluate orientation (e.g., age, date of birth, place, current year) and attention (e.g., list the months in reverse order). The 4AT has been validated for clinical use, with a sensitivity of 89.7% and specificity of 84.1% when compared to a psychiatric diagnosis of delirium [34]. The 4AT has been validated for use in assessing older adults [34–36]. The HABAM is a clinical tool used to assess patient in-bed mobility, transfers, and ambulation [37]. Given the sensitivity of HABAM dynamics to important clinical outcomes in hospital [38], more recently, it has been used to track the course of delirium, as it may offer a more stable estimate than does attention [39–41]. The HABAM has been used in geriatric medicine patients in both inpatient and outpatient settings but not explicitly in LTC homes [42].

The incidence of erythema and photokeratitis is collected via daily routine physical exams conducted by LTC facility staff.

If a resident tests positive for an RVI, their vital signs are collected daily via routine physical exam until they recover or die. All-cause mortality is collected from medical records by research nurses. Data collection in Phase 2 is unchanged from Phase 1.

### Plans to promote participant retention and complete follow-up {18b}

Data are collected during routine clinical care assessments.

### Data management {19}

Data are input to a REDCap database by the research nurses. The database is managed by a data management expert affiliated with the provincial health authority. Point of entry data validation mechanisms and systematic data checking algorithms are applied to ensure data quality standards.

### Confidentiality {27}

Numeric participant codes substitute for personal identifiers on research documents. All identifiable participant information is kept in locked cabinets and in password-protected computer files. Confidentiality of participants will be maintained in all forms of results reporting. Participants will be informed in general terms of the results as soon as is practical.

### Plans for collection, laboratory evaluation, and storage of biological specimens for genetic or molecular analysis in this trial/future use {33}

Nasopharyngeal swabs are the only biological specimens collected. These will not be saved.

## Statistical methods

### Statistical methods for primary and secondary outcomes {20a}

#### Descriptive analysis

Means, proportions, and medians will be produced with corresponding measures of variability (standard deviation and interquartile range) for key patient characteristics for the whole population and stratified by treatment assignment, neighborhood clusters, and site. Stage-wise heterogeneity in the descriptive statistics before and after adaption of the new lamps will also be explored. Kaplan–Meier curves will be used to illustrate trends in infection probabilities and a log-rank test will be used to estimate the crude difference in probability of RVI between the treated and control group, stratified by neighborhood clusters and site.

#### Primary analysis

The primary analysis will be an intention-to-treat effect estimated via comparison of 3-year incidence of COVID-19 or RVIs among individuals assigned to each treatment group. We will use a Cox model, which has the flexibility to account for the clustered nature of the data and adjust for prognostic factors, to estimate the hazard ratio. We will account for clustering within neighborhoods and by site using mixed-effects models [43]. Prognostic factors, at the individual (i.e., age, sex and frailty) will be explored and potentially adjusted for [44]. It is recommended that cluster-level prognostic factors are also adjusted for incluster randomized trials; however, we do not anticipate much variation here.

The change in lamps in Phase 2 present some unique analytical challenges. Participants in Phase 2 will presumably experience higher levels of far-UVC exposure. We will explore whether infection rates change between the two phases and will likely add a binary indicator to divide the person-time contributed into Phase 1 and Phase 2.

#### Person-time in study

Time zero will be defined by the date the lamps are turned on for all those enrolled at the beginning of the trial. For those enrolled subsequently, time zero is the date of consent. The person is followed until they are diagnosed with COVID-19 or other RVI, die, the study concludes, or participant withdrawal (administrative censoring). If a participant leaves the LTC facility for more than 24 h (e.g., is hospitalized), that person-time is excluded from the analysis. Participants who leave the LTC facilities may have an increased risk of infection. We will perform a sensitivity analysis where we introduce a washout period for all infections contracted 2 weeks after an absence from the home of more than 24 hours.

#### Secondary analysis

To assess the inter-rater reliability of the novel RVI screening protocol, prior to the trial, two research nurses assessed every participant once every 7 days, and concordance between raters was tracked. We will explore inter-rater agreement of each component (SQiD, SQiF, SQiRM, 4AT, and HABAM) using several agreement coefficients and their 95% confidence intervals, including percent agreement, kappa, Fliess’s kappa, Gwet’s AC, and Krippendorff’s alpha coefficient. Analysis will be carried out using Stata package *kappaetc* which follows methods and formulas discussed in Gwet [45]. We will also look at the sum of scores for SQiD, SQiF, and SQiRM and determine agreement for the overall score using the above measure and possibly ICC using a two-way mixed-effects model, provided model assumptions are met.

To assess the validity of the novel RVI screening protocol, the sensitivity, specificity, positive predictive value, and negative predictive value will be compared to the standard RVI screening protocol. Additionally, concordance between answers on the SQiD, SQirM, SQiF, 4AT, and HABAM will be tracked to determine if any questions are capturing otherwise undetected information.

#### Cost-consequence analysis

A cost-consequence analysis will be performed from a Canadian single-payer perspective. This will include assessing the purchasing, installation, and maintenance costs associated with the far-UVC lights; testing and treatment costs for RVIs, photokeratitis, and erythema; and the effects (consequences) associated with the intervention compared to the alternative. Outcome and patient characteristics data will come from the trial. Testing and treatment costs per case associated with the outcomes, including adverse outcomes, will come from the Canadian Institute for Health Information patient cost estimator [46], complemented with cost data from the literature. The analysis will involve estimating risk differences associated with RVIs, photokeratitis, and erythema between the intervention and control groups, using the augmented-inverse probability of treatment weighting models with a lasso for variable selection. Sensitivity analysis will be performed using a generalized linear model for the binomial family. Differences in mean testing and treatment costs between the two groups will be estimated using a two-part model.

Statistical analysis in Phase 2 is unchanged from Phase 1.

### Interim analyses {21b}

There are no interim analyses planned to preserve power of the final analysis.

### Methods for additional analyses {20b}

There are no subgroup analyses planned.

### Methods in analysis to handle protocol non-adherence and any statistical methods to handle missing data {20c}

The nature of the intervention is such that non-adherence is infeasible.

### Plans to give access to the full protocol, participant level-data and statistical code {31c}

The datasets analyzed during the current study and statistical code are available from the corresponding author on reasonable request, as is the full protocol.

## Oversight and monitoring

### Composition of the coordinating center and trial steering committee {5d}

Research coordinators at the Nova Scotia Health Geriatric Medicine Research team oversee the day-to-day running of the trial. The Scientific Advisory Council for this trial monitors data quality and safety of the study protocols with quarterly meetings. The Scientific Advisory Council is composed of representatives from the Nova Scotia government Department of Seniors and Long-Term Care, the Nova Scotia government Department of Public Health, the Dalhousie University Department of Civil and Resource Engineering, and the Dalhousie University Division of Infectious Diseases.

There is no discrete Stakeholder and Public Involvement Group. However, monthly newsletters are sent to LTC home residents and their family members to provide updates about the study. Each newsletter contains a survey to solicit feedback about residents’ experiences with study participation.

### Composition of the data monitoring committee and its role and reporting structure {21a}

Data quality is monitored by three parties: the main study coordinator, independent consultant biostatisticians, and a biostatistician on the Geriatric Medicine Research team who is otherwise not involved in the study.

### Adverse event reporting and harms {22}

All adverse events reported by participants or observed by the research team are recorded. Based on available data, there are theoretical risks of photokeratitis and skin erythema, but the dose and duration of exposure to UVC light make these risks minimal. Participants are screened daily for adverse events. If any significant adverse events occur, they are addressed immediately by the participant’s medical team and reported to the Scientific Advisory Committee and the Research Ethics Board to determine whether changes to the protocol need to be made.

### Frequency and plans for auditing trial conduct {23}

All research conducted at Nova Scotia Health is monitored and can be reviewed by Research Ethics Board auditors at any time.

### Plans for communicating important protocol amendments to relevant parties (e.g. trial participants, ethical committees) {25}

The research team has and will continue to keep participants, their substitute decision-makers, and LTC facility staff up to date about changes to the trial protocol with regular newsletters. Any amendments to the trial protocol will be submitted for approval by the Nova Scotia Health Research Ethics Board.

### Dissemination plans {31a}

Knowledge translation will include media engagement, peer-reviewed presentations, and publications of trial results.

## Ethics

### Ethics approval and consent to participate {24}

The protocol has been approved by the Nova Scotia Health Research Ethics Board. In this study, LTC facility residents are research participants because (1) they are exposed to study procedures: far-UVC light and the RVI screening protocol, and (2) their private identifiable data are collected. LTC facility staff and visitors are exposed to the intervention but cannot feasibly be randomized. Therefore, their data are not collected.

The far-UVC light intervention qualifies for an alteration of consent because it poses minimal risk and it is a cluster-level intervention, making it infeasible for LTC residents to decline exposure [47]. As a form of notification, regular newsletters disclosing the details of the study are sent to LTC facility stakeholders (residents, staff, and family caregivers), and posters describing the study are in common areas within the LTC facilities.

Researchers seek informed consent for the data collection from residents or their SDM. In lieu of formal capacity assessments, existing medical records of clinical decision-making capacity will be consulted. If a patient has documented decision-making capacity for clinical decisions, informed consent for data collection is sought from them directly. If they did not have clinical decision-making capacity, their SDM is approached to seek surrogate consent for data collection. This method of capacity assessment is appropriate because data collection procedures in this trial are akin to procedures in routine care [48].

## Discussion

The effectiveness of standard infection control measures is suboptimal in LTC facilities. If effective, far-UVC light could provide a valuable layer of protection from RVIs for LTC facility residents.

### Pragmatism

**Fig. 2:**
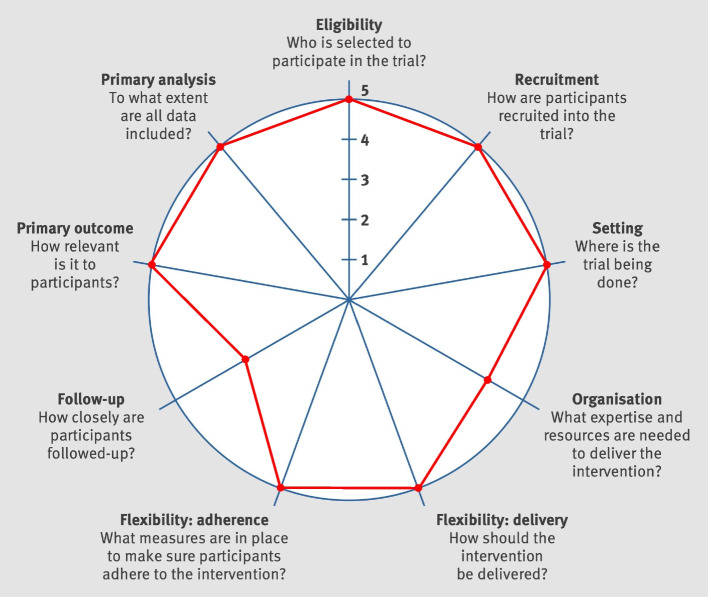
PRECIS-2 diagram analyzing trial design features on the explanatory-pragmatic spectrum

This trial has pragmatic intent [49], in that it aims to inform clinical decisions of whether far-UVC light should be implemented in LTC facilities to reduce the incidence of RVIs. Analyzing the features trial design using the PRagmatic Explanatory Continuum Indicator Summary (PRECIS)-2 tool demonstrates that the trial is highly pragmatic (Fig. 2) [50]. Trial eligibility criteria, recruitment, setting, flexibility (delivery), and flexibility (adherence) are highly pragmatic because they are consistent with how the intervention is intended to be used in clinical practice. The primary outcome is highly pragmatic because it is highly clinically relevant. The primary analysis is highly pragmatic because it is intention to treat. Organization and follow-up are rather pragmatic, because the presence of additional research nurses and additional monitoring of vitals in infected patients are slight deviations from routine clinical practice. Overall, the design of the trial is consistent with the pragmatic goal to produce evidence to inform a clinical decision. It conforms too with recommendations to broaden the evaluation of frailty status in relation to pandemic illness [51, 52], and understanding vaccine effectiveness in the common and at-risk population of older people who live with frailty [53], especially those who reside in LTC facilities [54].

### Vulnerabilities

LTC residents are commonly identified as a potentially vulnerable population of research participants [55]. Nix and colleagues developed a framework to identify and mitigate vulnerabilities arising in cluster randomized trials in LTC facilities [48]. Applying the framework demonstrates that the implemented additional ethical protections appropriately addressed each vulnerability (Table 2). Further, the ethical protections of gatekeeper permission and stakeholder engagement helped integrate research procedures into the clinical setting, promoting the pragmatic goals of the trial [25].

**Table 2:** Vulnerabilities and corresponding additional ethical protections

Ethical principle	Vulnerability	Additional ethical protection
*Autonomy*	Inadequate understanding in informed consent	Records of clinical decision-making capacity consulted in lieu of formal research capacity assessments Surrogate consent obtained from SDMs for residents lacking decision-making capacity
	Inadequate voluntariness in informed consent	Informed consent obtained by researchers with LTC facility administrators present, to make clear that participation is voluntary
	Invasion of privacy	Exposure to the intervention only in common areas Data collection procedures in residents’ bedrooms performed by LTC facility staff, as part of routine care Stakeholder engagement with pre-trial interviews and ongoing newsletters and surveys
*Welfare*	None—the study intervention and data collection procedures pose no more than minimal risk to participants	Not applicable
*Justice*	Unjust impact on care of nonparticipants	Gatekeeper permission obtained from LTC facility administrators Stakeholder engagement with pre-trial interviews and ongoing newsletters and surveys

### Limitations

A potential challenge is that LTC facility staff and visitors cannot feasibly be randomized. This poses a risk of contamination between study arms. However, there is some evidence to suggest that far-UVC light devices provide effective disinfection even as viral particles are continuously introduced into a space [16]. For this intervention, real-world effectiveness includes the ability to eliminate viral particles introduced into LTC facilities from visitors and staff who enter the space transiently. This potential contamination might hinder the internal validity of the trial, but it could simultaneously promote external validity.

The limited number of clusters and uncertainty in the survival probability have already resulted in challenges with determining of the appropriate sample size. We hope to have addressed the issue by adding an additional site and extending the study period; however, this will need to be re-examined at the 2-year timepoint, and it may be that additional sites will need to be added.

Another potential limitation is that far-UVC devices are not installed in resident bedrooms. This could hinder the effectiveness of the intervention, especially in bedrooms shared by two residents. However, limiting the intervention to common areas is justified by safety and ethical considerations. It mitigates the risk of adverse effects because (1) the far-UVC light devices emit a dose that is minimal risk for 8 h of exposure per day, which would be exceeded if residents were exposed overnight, and (2) the risk of photokeratitis is theoretically higher when lying supine, as residents are likely to do in their bedrooms. Further, limiting the intervention to common areas promotes residents’ privacy interests [48]. Finally, at current pricing, it is not financially feasible to obtain enough far-UVC devices to cover all bedrooms without industry support, which we have declined.

## Trial status

The current protocol is version 3, with an addendum for Phase 2. Protocol version 3 received approval from the Nova Scotia Health Research Ethics Board on September 9, 2021. The Phase 2 addendum received approval on December 21, 2022. Recruitment for Phase 1 of the trial began October 1, 2021. Recruitment for Phase 2 of the trial began on April 24, 2023. Recruitment for Phase 2 will continue until the end of the trial, April 2025.

## Conclusion

Far-UVC light offers a low maintenance, passive intervention to mitigate the spread of RVIs in LTC facilities. The PRiVII trial is the first randomized controlled trial of this intervention. The trial’s strong rationale, pragmatic design, and ethical protections will allow us to answer this socially valuable question while protecting the rights and welfare of participants.

### Supplementary Information

**Additional file 1. **Sample Size Estimation.

**Additional file 2. **Informed consent documents.

## Data Availability

The Scientific Advisory Council for this trial will monitor data quality and safety of the study protocols with quarterly meetings. Any data they require to support the protocol is made available to them on request.

## Abbreviations

4AT: 4 A’s test
ACGIH: American Conference of Governmental Industrial Hygienists
ANSI: American National Standards Institute
Far-UVC: Ultraviolet-C light at a wavelength of 222 nm
HABAM: Hierarchical Assessment of Balance and Mobility
ICC: Intra-class correlation coefficient
IES: Illuminating Engineering Society
LTC: Long-term care
PRECIS: PRagmatic Explanatory Continuum Indicator Summary
PRiVII: Preventing Respiratory Viral Illness Invisibly
RVI: Respiratory viral illness
SDM: Substitute decision maker
SQiD: Single question in delirium
SQiF: Single question in function
SQiRM: Single question in reduced mobility
TLV: Threshold Limit Value
