# Supplementary material for: Preventing Respiratory Viral Illness Invisibly (PRiVII): protocol for a pragmatic cluster randomized trial evaluating far-UVC light devices in long-term care facilities to reduce infections
Source: Trials. 2024 Jan 26;25:88. doi: 10.1186/s13063-024-07909-0 (PMC10811883; doi:10.1186/s13063-024-07909-0)
Supplement: Supplementary file 1 — Additional file 1. Sample Size Estimation. [file 13063_2024_7909_MOESM1_ESM.docx]

**Additional File 1.**

**Sample Size Estimation**

The sample size was determined using a log-rank test comparing two survival rates in a cluster randomized design. We have modelled several scenarios where we vary the survival probability between 0.4 and 0.7. The fixed parameters used were power at 0.8, alpha at 0.05, the number of clusters in the experimental and placebo groups (3 in control and 3 in the treatment) and a hazard ratio of 0.7. Analysis was completed using the Stata’s *power logrank, cluster* command.

Code: power logrank (0.4 0.5 0.6 0.7) , alpha(0.05) k1(3) k2(3) hratio (0.7) cvcluster(.1) rho(0) table(N E hratio s1 s2)

The line graph depicts the size of the control group cluster (M2) at different control group survival probabilities. With a 1:1 allocation ratio and 6 clusters in total, the total sample size needed (N) is equal to M2*6. In the table, we have total sample size needed (N), the number of events needed (E), the hazard ratio we want to detect (hratio), the survival probability in the control group (s1) and experimental group (s2). Without accounting for the ICC, we would require between 470 and 968 participants if the survival probability in the control group was 0.4 or 0.7 respectively.
