# Supplementary material for: Preventing Respiratory Viral Illness Invisibly (PRiVII): protocol for a pragmatic cluster randomized trial evaluating far-UVC light devices in long-term care facilities to reduce infections
Source: Trials. 2024 Jan 26;25:88. doi: 10.1186/s13063-024-07909-0 (PMC10811883; doi:10.1186/s13063-024-07909-0)
Supplement: Supplementary file 2 — Additional file 2. Informed consent documents. [file 13063_2024_7909_MOESM2_ESM.pdf]

nova scotia health authority

Informed Consent Form – *Long-Term Care Residents* *(main study)*

STUDY TITLE: Do far-UVC light devices reduce the incidence of influenza-like illnesses, respiratory illnesses, and COVID-19 infections in long-term care facilities?

CLINICAL TRIALS STUDY REGISTRATION NUMBER: NCT05084898

PRINCIPAL INVESTIGATOR: Dr. Kenneth Rockwood
Division of Geriatric Medicine, QEII Health Sciences Center
5955 Veterans' Memorial Lane, Halifax, NS B3H 2E1
902-473-8631

STUDY SPONSOR: Dr. Kenneth Rockwood

FUNDER: This study is being funded by Nova Scotia Health and the Department of Health and Wellness

---

<u>Substitute Decision Makers</u>: *You are reading this consent form on behalf of another person to decide if they will take part in this study. The words "*you*" and "*your*" in this document refer to that person.*

1. Introduction

You have been invited to take part in a research study. A research study is a way of gathering information on a treatment, procedure, or medical device or to answer a question about something that is not well understood. Taking part in this study is voluntary. It is up to you to decide whether you want to be in the study or not. Before you decide, you need to understand what the study is for, what risks you might take and what benefits you might receive. This consent form explains the study.

The research team will tell you if there are any study timelines for making your decision.

Please ask the research team to clarify anything you do not understand or would like to know more about. Make sure all your questions are answered to your satisfaction before deciding whether to participate in this research study.

The researchers will:
- Discuss the study with you
- Answer your questions
- Be available during the study to deal with problems and answer questions

You are being asked to consider participating in this study because you are a resident in long-term care at Northwood, Windsor Elms Village, or The Cove Guest Home.

If you decide not to take part or if you leave the study early, your usual health care will not be affected.

2. Why is there a need for this study?

Elderly people who have multiple health problems are at higher risk of illness from viral respiratory infections, such as influenza (the flu) and COVID-19. This is especially true for residents in long-term care because the usual methods of infection control (handwashing, mask-wearing, and distancing) are difficult to enforce due to the memory problems of many residents and the frequently shared common spaces.

It can also be difficult to prevent the spread of viral infections within long-term care because many residents are unable to tell their caregivers when they are feeling ill. Also, some elderly people do not show typical symptoms of infection (like fever), instead they may suddenly become confused or weak.

3. What is being tested?

In this study, we hope to reduce the number of respiratory infections among residents of long-term care through the use of safe low-dose ultraviolet light that is able to kill viruses living in the air and on surfaces, like walls and tables. Lamps that produce this light will be installed in the ceilings of common spaces shared by many residents (such as dining areas and activity rooms) and will be an extra method of disinfection in addition to the usual manual cleaning that is already being done.

The lamps that we will use in this study are called Zener, which are made by a Canadian company called UVX Incorporated. They do not give off any heat and the invisible light they produce is called far-ultraviolet C (or far-UVC), which is a form of ultraviolet light that has an extremely short wavelength (207-222 nm). Far-UVC light is strong enough to kill viruses in the air and on surfaces, but not strong enough to damage a person's skin or eye cells. (This is different from the other types of ultraviolet light which can cause damage, such as sunburn or skin cancer, with long or repeated exposures.) Research tests have shown that the far-UVC light that will be used in this study is not harmful to people, but to be extra safe, residents and staff will be monitored for unexpected side-effects.

Also, as part of this study, we hope to learn more about detecting the early signs of respiratory infections in elderly people by using daily health assessments to identify changes in mental and physical function.

4. How long will I be in this study?

The length of this study for participants is approximately 24 to 48 months (2 to 4 years), which will include at least two "flu seasons". The entire study is expected to take 3.5 years and the results should be known in 4 years.

5. How many people will take part in this study?

It is anticipated that up to 800 people could participate in this study in Nova Scotia. About 400 may participate at Northwood (Halifax), about 200 may participate at Windsor Elms Village (Falmouth), and an additional 200 may participate at the Cove Guest Home (Sydney).

6. How is the study being done

This is a placebo-controlled study. A placebo is an inactive treatment that looks the same as the active treatment that is being studied. A placebo is used to eliminate bias in the study, making the results of the study more reliable. In this study, the placebos will be lamps that look exactly like the active study lamps, but they will produce regular fluorescent light instead of far-UVC light. This fluorescent light will look the same as the far-UVC light, but it will have no effect on viruses.

The long-term care facility where you live has already decided that it will participate in this study and that your "neighbourhood" (living area of 30-36 residents) will have study lamps installed in the common spaces. There will be four study neighbourhoods at Northwood, two at Windsor Elms Village, and five at The Cove Guest Home.

At each site, the neighbourhoods will be randomly (by chance) assigned to be in one of two groups, active far-UVC light or placebo fluorescent light. This means your living area has a 50% chance of being assigned to either group. Neither you, the study staff, nor the investigators can influence or will know which group that will be. However, in case of an emergency the type of light in your neighbourhood can be identified.

7. What will happen if I take part in this study?

If you agree to participate and sign this consent form, a study nurse will review your health chart before the study lights are turned on and collect background information about you, such as:

- Demographics – e.g. age, sex, gender, ethnicity, and education/employment background
- Social information – e.g. how often you have visitors or phone calls, and if you have a roommate
- Care requirements – e.g. therapies and daily living assistance
- Vital measurements – e.g. height, weight, blood pressure, heart rate, and temperature
- Past health – e.g. vaccination records, previous viral infections, and past hospitalizations
- Current health – e.g. physical, mental, and emotional status

After the study lights have been turned on, your daily care team will notify the study nurse if they see a decline in your physical or mental function. For example, if you are having more trouble with your balance or ability to move around, or if you are less alert or seem more confused than the day before. The study nurse will then use routine clinical assessments to evaluate you further and measure any level of change in more detail. They will do these assessments daily, until you have seven days without more change. If one of these daily assessments confirms a decline in function that is large enough to be significant, the study nurse will collect a nasal swab to test for a possible respiratory virus infection (RVI swab).

A nasal swab is done by placing a small swab into the back of your nose for 5-10 seconds and rotating it gently before removal. Nasal swabs may already be a part of your usual health care or be required as part of public health testing for COVID-19, but participation in this study might mean you have extra swabs collected.

If you have a nasal swab that is positive for a respiratory virus infection, the study nurse will monitor your recovery and record daily vital measurements. If any public health precautions are required, like isolation, they will be followed.

8. Are there risks to this study?

There are risks with this study, as with any study. To give you the most complete information available, we have listed some *possible* risks. We do not want to alarm you, but we do want to make sure that you have had a chance to think about the risks carefully if you decide to participate in the study. Please be aware that there may be risks in participating in this study that we do not know about yet.

<u>Far-UVC light</u>

We do not expect there to be any side effects from your exposure to the far-UVC light. However, for extra safety, you will be monitored for side effects that are commonly caused by exposure to other forms of UV light (such as UVA and UVB from the sun). We will look for signs of damage to skin and eyes, like redness and pain.

RVI swabs
Nasal swab collection can cause slight discomfort and usually causes a person's eyes to water. You may also feel the need to cough. Rarely, a person can have a bloody nose.

Breach of confidentiality
As with all research, there is a chance that confidentiality could be compromised; however, we are taking steps to minimize this risk. To protect your personal information, a code number will be used to identify you during the study, and files that link your name to the code number will be kept in a secure place.

9. Are there benefits of participating in this study?

You may or may not benefit directly from participating in this study. Your participation may or may not help other people with who live in long-term care in the future.

10. Are there other choices?

If you decide not to participate in this study, your personal information will not be collected and you will not have any study assessments done.

11. What happens at the end of this study?

We expect that the results from this study will be published and presented in a variety of forums. In any publication and/or presentation, information will be provided in such a way that you cannot be identified, except with your express permission.

12. What are my responsibilities?

As a study participant you will be expected to:

- Follow the directions of the research team;
- Report any problems you experience that you think might be related to participating in the study.

13. Can my participation in this study end early?

Yes. The Nova Scotia Health Research Ethics Board and the principal investigator have the right to stop patient recruitment or cancel the study at any time.

Also, the principal investigator may decide to remove you from this study without your consent for any of the following reasons:

- You do not follow the directions of the research team;
- There is new information that shows that being in this study is not in your best interest.

If you are withdrawn from this study, a member of the research team will discuss the reasons with you and it will have no effect on your continued care outside the study.

You can also decide to end your participation at any time and this will not affect your current or future medical treatment and healthcare. If you choose to withdraw your consent, please inform the research team. Data collected up to this point will still be included in the study analysis.

14. What about new information?

You will be told about any other new information that might affect your health, welfare, or willingness to stay in the study and will be asked whether you wish to continue taking part in the study or not.

15. Will it cost me anything?

Compensation

Participation in this study will not involve any additional costs to you.

Research Related Injury

If you become ill or injured as a direct result of participating in this study, necessary medical treatment will be available at no additional cost to you. Your signature on this form only indicates that you have understood to your satisfaction the information regarding your participation in the study and agree to participate in the study. In no way does this waive your legal rights nor release the principal investigator, the research team, the study sponsor or involved institutions from their legal and professional responsibilities.

16. What about my privacy and confidentiality?

Protecting your privacy is an important part of this study and every effort to protect your privacy will be made. However, complete privacy cannot be guaranteed. For example, the principal investigator may be required by law to allow access to research records.

If the results of this study are presented to the public, nobody will be able to tell that you were in the study.

If you decide to participate in this study, the research team will collect personal health information from you and your health record. The research team will collect and use only the information they need for this study and to judge the safety and usefulness of the study treatment.

"Personal health information" is health information about you that could identify you because it includes information such as your;

- Name,
- Age or month/year of birth (MM/YY),
- New and existing medical records, or
- The types, dates and results of various tests and procedures.

Access to Records

Other people may need to look at your personal health information to check that the information collected for the study is correct and to make sure the study followed the required laws and guidelines. These people might include:

- The Nova Scotia Health Research Ethics Board (NS Health REB) and people working for or with the NS Health REB because they oversee the ethical conduct of research studies within Nova Scotia Health;

- When you sign this consent form you give us permission to collect information from the Department of Health and Wellness' SHARE Electronic Health Record which contains laboratory test results, Diagnostic Imaging, as well as admissions, discharge and transfer records from across the province.

These people will view your study records at this institution, either remotely through secure access or in person, and will not take identifying information away with them.

Use of Your Study Information

Any study data about you that is sent outside of Nova Scotia Health will have a code and will not contain your name or address, or any information that directly identifies you. De-identified study data may be transferred to:

- Regulatory authorities within and outside Canada.

Study data that is sent outside of Nova Scotia Health will be used for the research purposes explained in this consent form. De-identified data from this study may also be used to answer future research questions that may arise.

The research team and the other people listed above will keep the information they see or receive about you confidential, to the extent permitted by applicable laws. Even though the risk of identifying you from the study data is very small, it can never be completely eliminated.

The research team will keep any personal health information about you in a secure and confidential location for seven years after the study ends and then destroy it according to NS Health policy. Your personal health information will not be shared with others without your permission.

After your part in the study ends, we may continue to review your health records for safety and data accuracy until the study is finished or you withdraw your consent.

The REB and people working for or with the REB may also contact you personally for quality assurance purposes.

Your access to records

You have the right to access, review, and request changes to your study data. Since the study is "blinded," you cannot see this information until the study ends. This prevents either you or your doctor from knowing which study treatment you received until the results are reported.

17. Declaration of financial interest

The funder is reimbursing the principal investigator and/or the principal investigator’s institution to conduct this study. The amount of payment is sufficient to cover the costs of conducting the study.

18. What about questions or problems?

For further information about the study you may call the principal investigator, who is the person in charge of this study and/or any other research team member listed below.

The principal investigator is:	Dr. Kenneth Rockwood	902-473-8631
The study nurses at **Northwood** are:	Ann Mann & Rosemary Boettinger	902-454-8311 ext. 3118
The study nurse at **Windsor Elms Village** is:	Kim Langdon	902-798-2251 ext. 288
The study nurse at **The Cove Guest Hom**e is:	Hailey Reel	902-539-5267 ext. 230

19. What are my rights?

You have the right to all information to help you decide whether or not to participate in this study. You also have the right to ask questions about this study and to have them answered to your satisfaction before you make any decision. You also have the right to ask questions and to receive answers throughout this study. You have the right to withdraw your consent at any time.

If you have questions about your rights as a research participant and/or concerns or complaints about this research study, you can contact

1. The Nova Scotia Health Research Ethics Board Office
    - email: ResearchEthics@nshealth.ca
    - Phone: 902-222-9263
2. Patient Relations, Nova Scotia Health
    - Email: HealthCareExperience@nshealth.ca
    - Phone: 1-844-884-4177

20. Consent Form Signature Page

I have reviewed all the information in this consent form related to the study called:

Do far-UVC light devices reduce the incidence of influenza-like illnesses, respiratory illnesses, and COVID-19 infections in long-term care facilities?

I was given the opportunity to discuss this study and all my questions were answered to my satisfaction.

*Note for Substitute Decision Makers: If you are consenting to this study on behalf of another person, the words "*I*" and "*my*" in this section below refer to that person.*

I allow access to my personal health information and study data, as explained in this form.

This signature on this consent form means that I agree to take part in this study. I understand that I am free to withdraw at any time without affecting my future care.

____________________ Signature of Participant	____________________ Name (Printed)	______ / ______ / ______ Year Month Day*
____________________ Signature of Participant's Substitute Decision Maker	____________________ Name (Printed)	______ / ______ / ______ Year Month Day*
____________________ Signature of Person Conducting Consent Discussion	____________________ Name (Printed)	______ / ______ / ______ Year Month Day*
____________________ Signature of Investigator	____________________ Name (Printed)	______ / ______ / ______ Year Month Day*
____________________ Signature of Impartial Witness *(If needed)*	____________________ Name (Printed)	______ / ______ / ______ Year Month Day*

Note: Please fill in the dates personally

I will be given a signed copy of this consent form.

Thank you for your time and patience!
